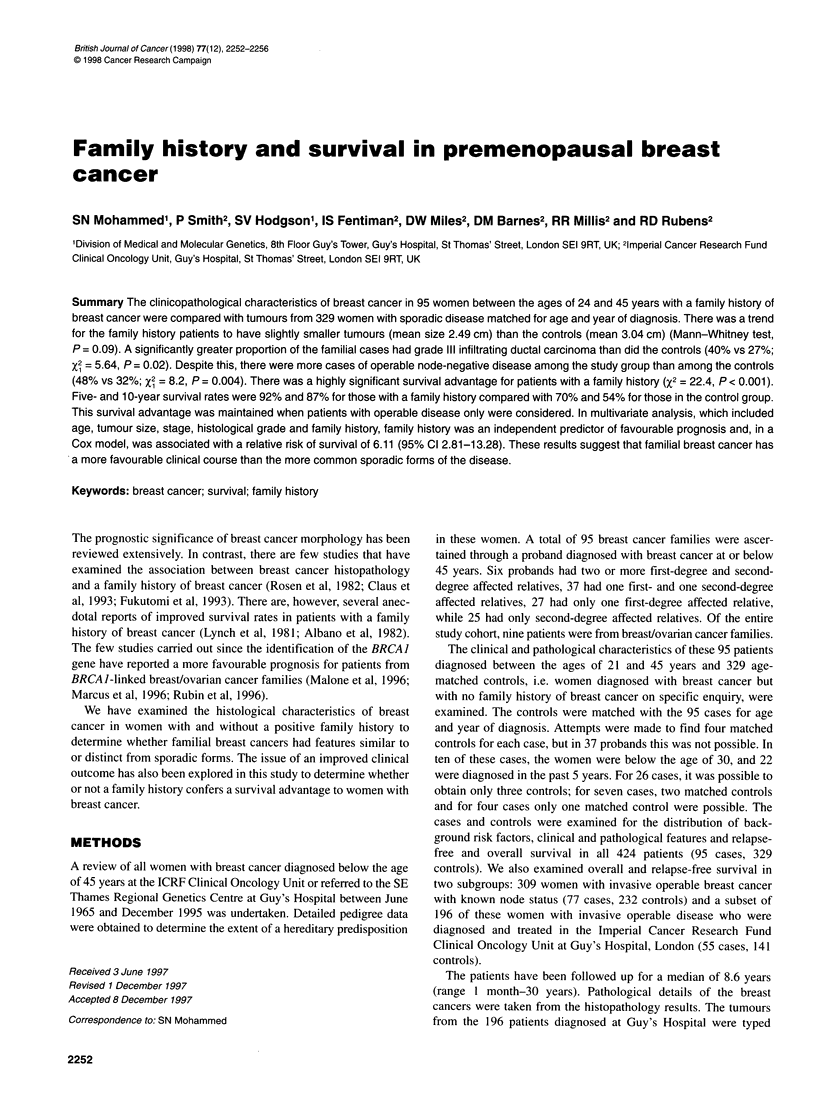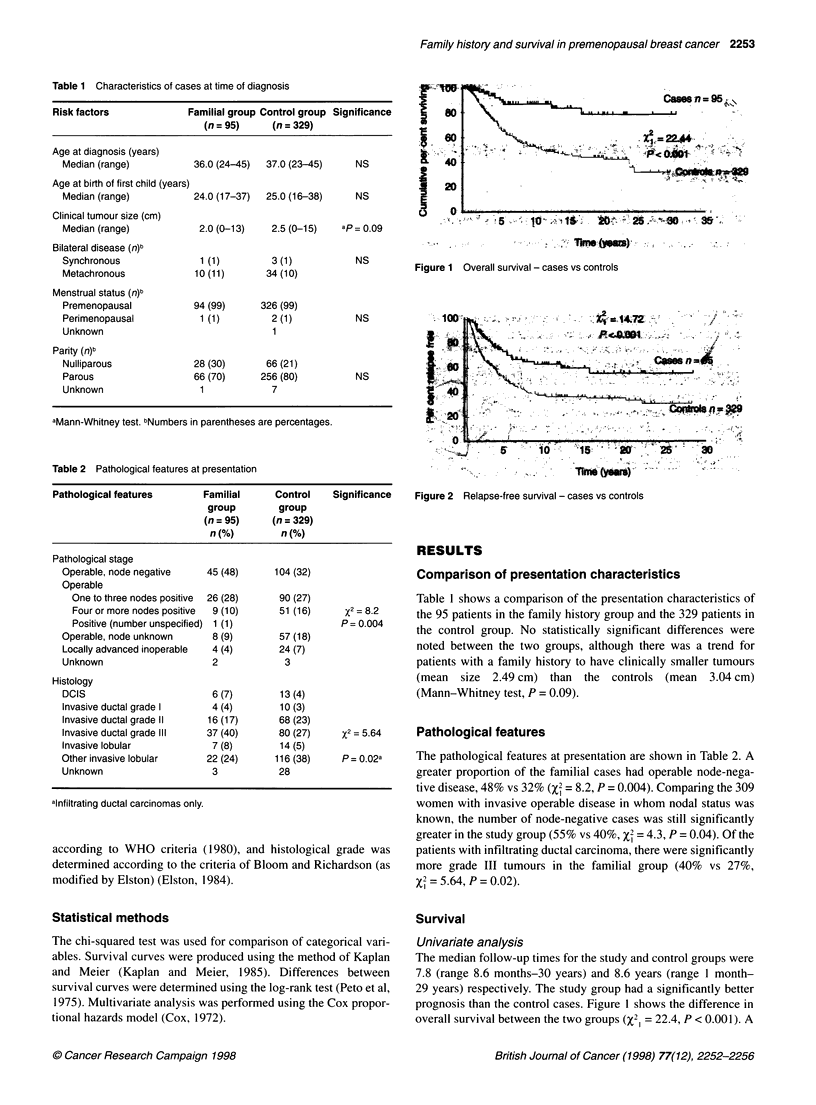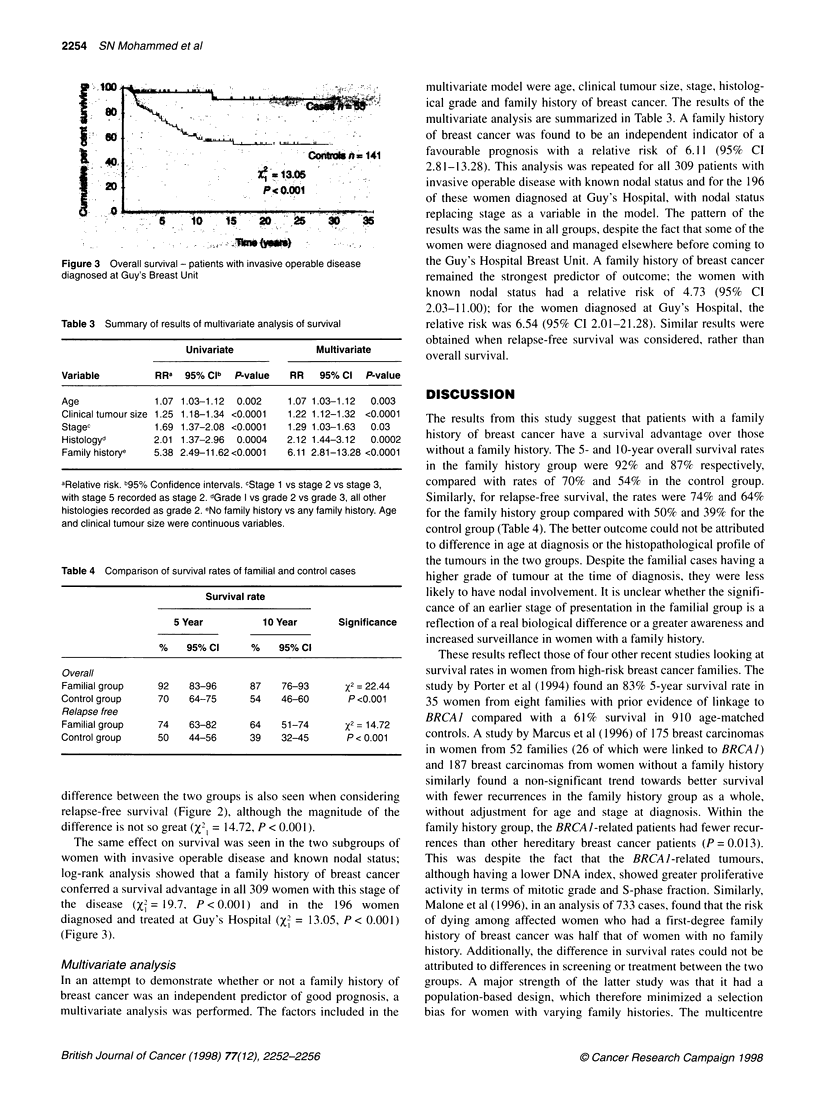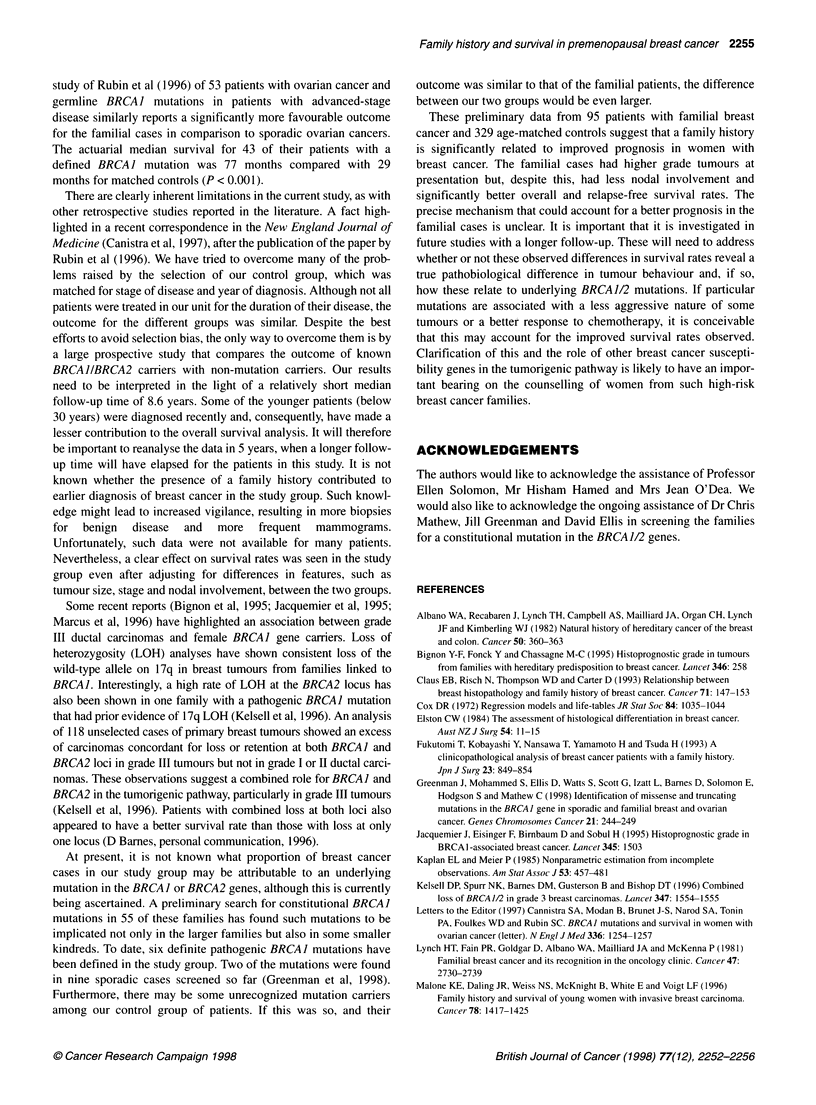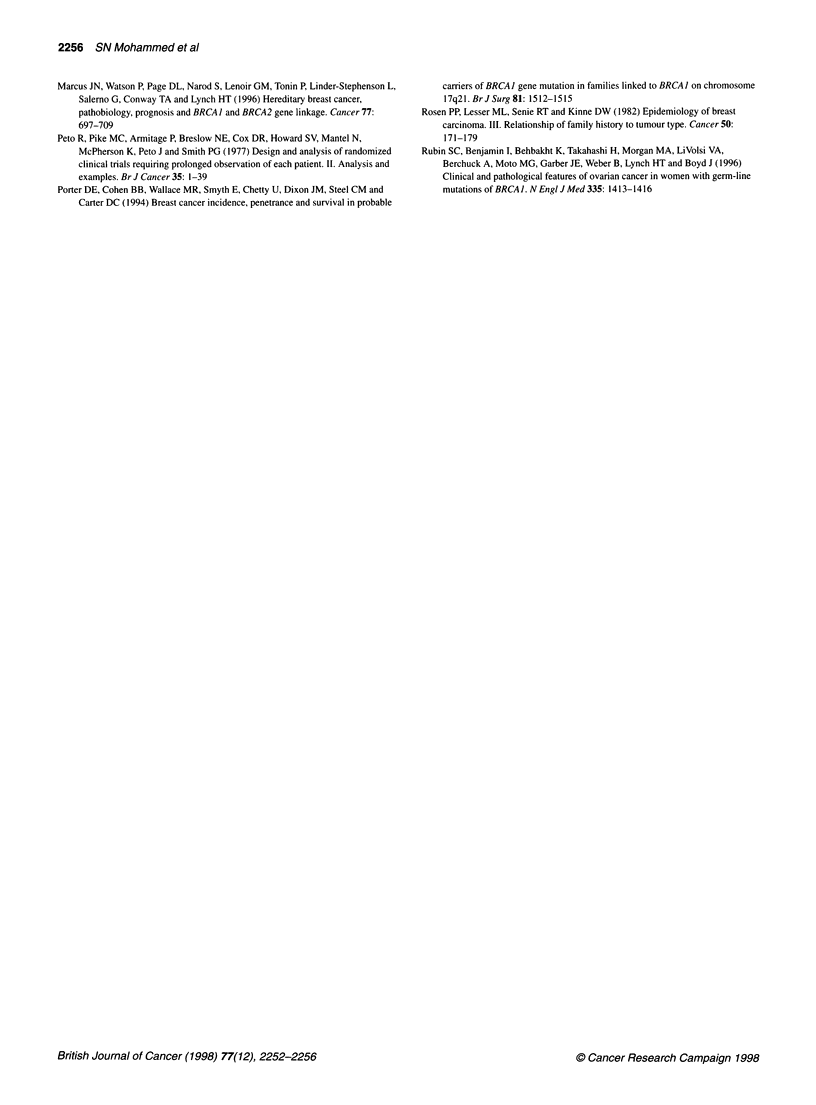# Family history and survival in premenopausal breast cancer.

**DOI:** 10.1038/bjc.1998.374

**Published:** 1998-06

**Authors:** S. N. Mohammed, P. Smith, S. V. Hodgson, I. S. Fentiman, D. W. Miles, D. M. Barnes, R. R. Millis, R. D. Rubens

**Affiliations:** Division of Medical and Molecular Genetics, Guy's Hospital, London, UK.

## Abstract

The clinicopathological characteristics of breast cancer in 95 women between the ages of 24 and 45 years with a family history of breast cancer were compared with tumours from 329 women with sporadic disease matched for age and year of diagnosis. There was a trend for the family history patients to have slightly smaller tumours (mean size 2.49 cm) than the controls (mean 3.04 cm) (Mann-Whitney test, P = 0.09). A significantly greater proportion of the familial cases had grade III infiltrating ductal carcinoma than did the controls (40% vs 27%; chi2(1) = 5.64, P = 0.02). Despite this, there were more cases of operable node-negative disease among the study group than among the controls (48% vs 32%; chi2(1) = 8.2, P = 0.004). There was a highly significant survival advantage for patients with a family history (chi2 = 22.4, P < 0.001). Five- and 10-year survival rates were 92% and 87% for those with a family history compared with 70% and 54% for those in the control group. This survival advantage was maintained when patients with operable disease only were considered. In multivariate analysis, which included age, tumour size, stage, histological grade and family history, family history was an independent predictor of favourable prognosis and, in a Cox model, was associated with a relative risk of survival of 6.11 (95% CI 2.81-13.28). These results suggest that familial breast cancer has a more favourable clinical course than the more common sporadic forms of the disease.


British Journal of Cancer (1998) 77(12), 2252–2256
© 1998 Cancer Research Campaign

### Family history and survival in premenopausal breast cancer

**SN Mohammed[1], P Smith[2], SV Hodgson[1], IS Fentiman[2], DW Miles[2], DM Barnes[2], RR Millis[2] and RD Rubens[2]**

[1]Division of Medical and Molecular Genetics, 8th Floor Guy's Tower, Guy's Hospital, St Thomas' Street, London SEI 9RT, UK; [2]Imperial Cancer Research Fund Clinical Oncology Unit, Guy's Hospital, St Thomas' Street, London SEI 9RT, UK

**Summary** The clinicopathological characteristics of breast cancer in 95 women between the ages of 24 and 45 years with a family history of breast cancer were compared with tumours from 329 women with sporadic disease matched for age and year of diagnosis. There was a trend for the family history patients to have slightly smaller tumours (mean size 2.49 cm) than the controls (mean 3.04 cm) (Mann–Whitney test, $P = 0.09$). A significantly greater proportion of the familial cases had grade III infiltrating ductal carcinoma than did the controls (40% vs 27%; $\chi^2_1 = 5.64$, $P = 0.02$). Despite this, there were more cases of operable node-negative disease among the study group than among the controls (48% vs 32%; $\chi^2_1 = 8.2$, $P = 0.004$). There was a highly significant survival advantage for patients with a family history ($\chi^2 = 22.4$, $P < 0.001$). Five- and 10-year survival rates were 92% and 87% for those with a family history compared with 70% and 54% for those in the control group. This survival advantage was maintained when patients with operable disease only were considered. In multivariate analysis, which included age, tumour size, stage, histological grade and family history, family history was an independent predictor of favourable prognosis and, in a Cox model, was associated with a relative risk of survival of 6.11 (95% CI 2.81–13.28). These results suggest that familial breast cancer has a more favourable clinical course than the more common sporadic forms of the disease.

**Keywords:** breast cancer; survival; family history

The prognostic significance of breast cancer morphology has been reviewed extensively. In contrast, there are few studies that have examined the association between breast cancer histopathology and a family history of breast cancer (Rosen et al, 1982; Claus et al, 1993; Fukutomi et al, 1993). There are, however, several anecdotal reports of improved survival rates in patients with a family history of breast cancer (Lynch et al, 1981; Albano et al, 1982). The few studies carried out since the identification of the *BRCA1* gene have reported a more favourable prognosis for patients from *BRCA1*-linked breast/ovarian cancer families (Malone et al, 1996; Marcus et al, 1996; Rubin et al, 1996).

We have examined the histological characteristics of breast cancer in women with and without a positive family history to determine whether familial breast cancers had features similar to or distinct from sporadic forms. The issue of an improved clinical outcome has also been explored in this study to determine whether or not a family history confers a survival advantage to women with breast cancer.

### METHODS

A review of all women with breast cancer diagnosed below the age of 45 years at the ICRF Clinical Oncology Unit or referred to the SE Thames Regional Genetics Centre at Guy's Hospital between June 1965 and December 1995 was undertaken. Detailed pedigree data were obtained to determine the extent of a hereditary predisposition in these women. A total of 95 breast cancer families were ascertained through a proband diagnosed with breast cancer at or below 45 years. Six probands had two or more first-degree and second-degree affected relatives, 37 had one first- and one second-degree affected relatives, 27 had only one first-degree affected relative, while 25 had only second-degree affected relatives. Of the entire study cohort, nine patients were from breast/ovarian cancer families.

The clinical and pathological characteristics of these 95 patients diagnosed between the ages of 21 and 45 years and 329 age-matched controls, i.e. women diagnosed with breast cancer but with no family history of breast cancer on specific enquiry, were examined. The controls were matched with the 95 cases for age and year of diagnosis. Attempts were made to find four matched controls for each case, but in 37 probands this was not possible. In ten of these cases, the women were below the age of 30, and 22 were diagnosed in the past 5 years. For 26 cases, it was possible to obtain only three controls; for seven cases, two matched controls and for four cases only one matched control were possible. The cases and controls were examined for the distribution of background risk factors, clinical and pathological features and relapse-free and overall survival in all 424 patients (95 cases, 329 controls). We also examined overall and relapse-free survival in two subgroups: 309 women with invasive operable breast cancer with known node status (77 cases, 232 controls) and a subset of 196 of these women with invasive operable disease who were diagnosed and treated in the Imperial Cancer Research Fund Clinical Oncology Unit at Guy's Hospital, London (55 cases, 141 controls).

The patients have been followed up for a median of 8.6 years (range 1 month–30 years). Pathological details of the breast cancers were taken from the histopathology results. The tumours from the 196 patients diagnosed at Guy's Hospital were typed

*Received 3 June 1997*
*Revised 1 December 1997*
*Accepted 8 December 1997*

*Correspondence to:* SN Mohammed

**Table 1** Characteristics of cases at time of diagnosis

| Risk factors | Familial group (*n* = 95) | Control group (*n* = 329) | Significance |
|---|---|---|---|
| Age at diagnosis (years) | | | |
| Median (range) | 36.0 (24–45) | 37.0 (23–45) | NS |
| Age at birth of first child (years) | | | |
| Median (range) | 24.0 (17–37) | 25.0 (16–38) | NS |
| Clinical tumour size (cm) | | | |
| Median (range) | 2.0 (0–13) | 2.5 (0–15) | [a]*P* = 0.09 |
| Bilateral disease (*n*)[b] | | | |
| Synchronous | 1 (1) | 3 (1) | NS |
| Metachronous | 10 (11) | 34 (10) | |
| Menstrual status (*n*)[b] | | | |
| Premenopausal | 94 (99) | 326 (99) | |
| Perimenopausal | 1 (1) | 2 (1) | NS |
| Unknown | | 1 | |
| Parity (*n*)[b] | | | |
| Nulliparous | 28 (30) | 66 (21) | |
| Parous | 66 (70) | 256 (80) | NS |
| Unknown | 1 | 7 | |

[a]Mann-Whitney test. [b]Numbers in parentheses are percentages.

**Table 2** Pathological features at presentation

| Pathological features | Familial group (*n* = 95) *n* (%) | Control group (*n* = 329) *n* (%) | Significance |
|---|---|---|---|
| Pathological stage | | | |
| Operable, node negative | 45 (48) | 104 (32) | |
| Operable | | | |
| One to three nodes positive | 26 (28) | 90 (27) | |
| Four or more nodes positive | 9 (10) | 51 (16) | $\chi^2 = 8.2$ |
| Positive (number unspecified) | 1 (1) | | $P = 0.004$ |
| Operable, node unknown | 8 (9) | 57 (18) | |
| Locally advanced inoperable | 4 (4) | 24 (7) | |
| Unknown | 2 | 3 | |
| Histology | | | |
| DCIS | 6 (7) | 13 (4) | |
| Invasive ductal grade I | 4 (4) | 10 (3) | |
| Invasive ductal grade II | 16 (17) | 68 (23) | |
| Invasive ductal grade III | 37 (40) | 80 (27) | $\chi^2 = 5.64$ |
| Invasive lobular | 7 (8) | 14 (5) | |
| Other invasive lobular | 22 (24) | 116 (38) | *P* = 0.02[a] |
| Unknown | 3 | 28 | |

[a]Infiltrating ductal carcinomas only.

according to WHO criteria (1980), and histological grade was determined according to the criteria of Bloom and Richardson (as modified by Elston) (Elston, 1984).

### Statistical methods

The chi-squared test was used for comparison of categorical variables. Survival curves were produced using the method of Kaplan and Meier (Kaplan and Meier, 1985). Differences between survival curves were determined using the log-rank test (Peto et al, 1975). Multivariate analysis was performed using the Cox proportional hazards model (Cox, 1972).

**Figure 1** Overall survival – cases vs controls

**Figure 2** Relapse-free survival – cases vs controls

### RESULTS

### Comparison of presentation characteristics

Table 1 shows a comparison of the presentation characteristics of the 95 patients in the family history group and the 329 patients in the control group. No statistically significant differences were noted between the two groups, although there was a trend for patients with a family history to have clinically smaller tumours (mean size 2.49 cm) than the controls (mean 3.04 cm) (Mann–Whitney test, $P = 0.09$).

### Pathological features

The pathological features at presentation are shown in Table 2. A greater proportion of the familial cases had operable node-negative disease, 48% vs 32% ($\chi_1^2 = 8.2$, $P = 0.004$). Comparing the 309 women with invasive operable disease in whom nodal status was known, the number of node-negative cases was still significantly greater in the study group (55% vs 40%, $\chi_1^2 = 4.3$, $P = 0.04$). Of the patients with infiltrating ductal carcinoma, there were significantly more grade III tumours in the familial group (40% vs 27%, $\chi_1^2 = 5.64$, $P = 0.02$).

### Survival

### *Univariate analysis*

The median follow-up times for the study and control groups were 7.8 (range 8.6 months–30 years) and 8.6 years (range 1 month–29 years) respectively. The study group had a significantly better prognosis than the control cases. Figure 1 shows the difference in overall survival between the two groups ($\chi^2{}_1 = 22.4$, $P < 0.001$). A

*© Cancer Research Campaign 1998* 

**Figure 3** Overall survival – patients with invasive operable disease diagnosed at Guy's Breast Unit

**Table 3** Summary of results of multivariate analysis of survival

| | Univariate | | | Multivariate | | |
|---|---|---|---|---|---|---|
| **Variable** | **RR[a]** | **95% CI[b]** | ***P*-value** | **RR** | **95% CI** | ***P*-value** |
| Age | 1.07 | 1.03–1.12 | 0.002 | 1.07 | 1.03–1.12 | 0.003 |
| Clinical tumour size | 1.25 | 1.18–1.34 | <0.0001 | 1.22 | 1.12–1.32 | <0.0001 |
| Stage[c] | 1.69 | 1.37–2.08 | <0.0001 | 1.29 | 1.03–1.63 | 0.03 |
| Histology[d] | 2.01 | 1.37–2.96 | 0.0004 | 2.12 | 1.44–3.12 | 0.0002 |
| Family history[e] | 5.38 | 2.49–11.62 | <0.0001 | 6.11 | 2.81–13.28 | <0.0001 |

[a]Relative risk. [b]95% Confidence intervals. [c]Stage 1 vs stage 2 vs stage 3, with stage 5 recorded as stage 2. [d]Grade I vs grade 2 vs grade 3, all other histologies recorded as grade 2. [e]No family history vs any family history. Age and clinical tumour size were continuous variables.

**Table 4** Comparison of survival rates of familial and control cases

| | Survival rate | | | | |
|---|---|---|---|---|---|
| | 5 Year | | 10 Year | | Significance |
| | **%** | **95% CI** | **%** | **95% CI** | |
| *Overall* | | | | | |
| Familial group | 92 | 83–96 | 87 | 76–93 | $\chi^2$ = 22.44 |
| Control group | 70 | 64–75 | 54 | 46–60 | $P$ <0.001 |
| *Relapse free* | | | | | |
| Familial group | 74 | 63–82 | 64 | 51–74 | $\chi^2$ = 14.72 |
| Control group | 50 | 44–56 | 39 | 32–45 | $P$ < 0.001 |

difference between the two groups is also seen when considering relapse-free survival (Figure 2), although the magnitude of the difference is not so great ($\chi^2_1 = 14.72$, $P < 0.001$).

The same effect on survival was seen in the two subgroups of women with invasive operable disease and known nodal status; log-rank analysis showed that a family history of breast cancer conferred a survival advantage in all 309 women with this stage of the disease ($\chi^2_1 = 19.7$, $P < 0.001$) and in the 196 women diagnosed and treated at Guy's Hospital ($\chi^2_1 = 13.05$, $P < 0.001$) (Figure 3).

### *Multivariate analysis*

In an attempt to demonstrate whether or not a family history of breast cancer was an independent predictor of good prognosis, a multivariate analysis was performed. The factors included in the multivariate model were age, clinical tumour size, stage, histological grade and family history of breast cancer. The results of the multivariate analysis are summarized in Table 3. A family history of breast cancer was found to be an independent indicator of a favourable prognosis with a relative risk of 6.11 (95% CI 2.81–13.28). This analysis was repeated for all 309 patients with invasive operable disease with known nodal status and for the 196 of these women diagnosed at Guy's Hospital, with nodal status replacing stage as a variable in the model. The pattern of the results was the same in all groups, despite the fact that some of the women were diagnosed and managed elsewhere before coming to the Guy's Hospital Breast Unit. A family history of breast cancer remained the strongest predictor of outcome; the women with known nodal status had a relative risk of 4.73 (95% CI 2.03–11.00); for the women diagnosed at Guy's Hospital, the relative risk was 6.54 (95% CI 2.01–21.28). Similar results were obtained when relapse-free survival was considered, rather than overall survival.

### DISCUSSION

The results from this study suggest that patients with a family history of breast cancer have a survival advantage over those without a family history. The 5- and 10-year overall survival rates in the family history group were 92% and 87% respectively, compared with rates of 70% and 54% in the control group. Similarly, for relapse-free survival, the rates were 74% and 64% for the family history group compared with 50% and 39% for the control group (Table 4). The better outcome could not be attributed to difference in age at diagnosis or the histopathological profile of the tumours in the two groups. Despite the familial cases having a higher grade of tumour at the time of diagnosis, they were less likely to have nodal involvement. It is unclear whether the significance of an earlier stage of presentation in the familial group is a reflection of a real biological difference or a greater awareness and increased surveillance in women with a family history.

These results reflect those of four other recent studies looking at survival rates in women from high-risk breast cancer families. The study by Porter et al (1994) found an 83% 5-year survival rate in 35 women from eight families with prior evidence of linkage to *BRCA1* compared with a 61% survival in 910 age-matched controls. A study by Marcus et al (1996) of 175 breast carcinomas in women from 52 families (26 of which were linked to *BRCA1*) and 187 breast carcinomas from women without a family history similarly found a non-significant trend towards better survival with fewer recurrences in the family history group as a whole, without adjustment for age and stage at diagnosis. Within the family history group, the *BRCA1*-related patients had fewer recurrences than other hereditary breast cancer patients ($P = 0.013$). This was despite the fact that the *BRCA1*-related tumours, although having a lower DNA index, showed greater proliferative activity in terms of mitotic grade and S-phase fraction. Similarly, Malone et al (1996), in an analysis of 733 cases, found that the risk of dying among affected women who had a first-degree family history of breast cancer was half that of women with no family history. Additionally, the difference in survival rates could not be attributed to differences in screening or treatment between the two groups. A major strength of the latter study was that it had a population-based design, which therefore minimized a selection bias for women with varying family histories. The multicentre

 © Cancer Research Campaign 1998

study of Rubin et al (1996) of 53 patients with ovarian cancer and germline *BRCA1* mutations in patients with advanced-stage disease similarly reports a significantly more favourable outcome for the familial cases in comparison to sporadic ovarian cancers. The actuarial median survival for 43 of their patients with a defined *BRCA1* mutation was 77 months compared with 29 months for matched controls ($P < 0.001$).

There are clearly inherent limitations in the current study, as with other retrospective studies reported in the literature. A fact highlighted in a recent correspondence in the *New England Journal of Medicine* (Canistra et al, 1997), after the publication of the paper by Rubin et al (1996). We have tried to overcome many of the problems raised by the selection of our control group, which was matched for stage of disease and year of diagnosis. Although not all patients were treated in our unit for the duration of their disease, the outcome for the different groups was similar. Despite the best efforts to avoid selection bias, the only way to overcome them is by a large prospective study that compares the outcome of known *BRCA1*/*BRCA2* carriers with non-mutation carriers. Our results need to be interpreted in the light of a relatively short median follow-up time of 8.6 years. Some of the younger patients (below 30 years) were diagnosed recently and, consequently, have made a lesser contribution to the overall survival analysis. It will therefore be important to reanalyse the data in 5 years, when a longer follow-up time will have elapsed for the patients in this study. It is not known whether the presence of a family history contributed to earlier diagnosis of breast cancer in the study group. Such knowledge might lead to increased vigilance, resulting in more biopsies for benign disease and more frequent mammograms. Unfortunately, such data were not available for many patients. Nevertheless, a clear effect on survival rates was seen in the study group even after adjusting for differences in features, such as tumour size, stage and nodal involvement, between the two groups.

Some recent reports (Bignon et al, 1995; Jacquemier et al, 1995; Marcus et al, 1996) have highlighted an association between grade III ductal carcinomas and female *BRCA1* gene carriers. Loss of heterozygosity (LOH) analyses have shown consistent loss of the wild-type allele on 17q in breast tumours from families linked to *BRCA1*. Interestingly, a high rate of LOH at the *BRCA2* locus has also been shown in one family with a pathogenic *BRCA1* mutation that had prior evidence of 17q LOH (Kelsell et al, 1996). An analysis of 118 unselected cases of primary breast tumours showed an excess of carcinomas concordant for loss or retention at both *BRCA1* and *BRCA2* loci in grade III tumours but not in grade I or II ductal carcinomas. These observations suggest a combined role for *BRCA1* and *BRCA2* in the tumorigenic pathway, particularly in grade III tumours (Kelsell et al, 1996). Patients with combined loss at both loci also appeared to have a better survival rate than those with loss at only one locus (D Barnes, personal communication, 1996).

At present, it is not known what proportion of breast cancer cases in our study group may be attributable to an underlying mutation in the *BRCA1* or *BRCA2* genes, although this is currently being ascertained. A preliminary search for constitutional *BRCA1* mutations in 55 of these families has found such mutations to be implicated not only in the larger families but also in some smaller kindreds. To date, six definite pathogenic *BRCA1* mutations have been defined in the study group. Two of the mutations were found in nine sporadic cases screened so far (Greenman et al, 1998). Furthermore, there may be some unrecognized mutation carriers among our control group of patients. If this was so, and their outcome was similar to that of the familial patients, the difference between our two groups would be even larger.

These preliminary data from 95 patients with familial breast cancer and 329 age-matched controls suggest that a family history is significantly related to improved prognosis in women with breast cancer. The familial cases had higher grade tumours at presentation but, despite this, had less nodal involvement and significantly better overall and relapse-free survival rates. The precise mechanism that could account for a better prognosis in the familial cases is unclear. It is important that it is investigated in future studies with a longer follow-up. These will need to address whether or not these observed differences in survival rates reveal a true pathobiological difference in tumour behaviour and, if so, how these relate to underlying *BRCA1/2* mutations. If particular mutations are associated with a less aggressive nature of some tumours or a better response to chemotherapy, it is conceivable that this may account for the improved survival rates observed. Clarification of this and the role of other breast cancer susceptibility genes in the tumorigenic pathway is likely to have an important bearing on the counselling of women from such high-risk breast cancer families.

### ACKNOWLEDGEMENTS

The authors would like to acknowledge the assistance of Professor Ellen Solomon, Mr Hisham Hamed and Mrs Jean O'Dea. We would also like to acknowledge the ongoing assistance of Dr Chris Mathew, Jill Greenman and David Ellis in screening the families for a constitutional mutation in the *BRCA1/2* genes.

### REFERENCES

Albano WA, Recabaren J, Lynch TH, Campbell AS, Mailliard JA, Organ CH, Lynch JF and Kimberling WJ (1982) Natural history of hereditary cancer of the breast and colon. *Cancer* **50**: 360–363

Bignon Y-F, Fonck Y and Chassagne M-C (1995) Histoprognostic grade in tumours from families with hereditary predisposition to breast cancer. *Lancet* **346**: 258

Claus EB, Risch N, Thompson WD and Carter D (1993) Relationship between breast histopathology and family history of breast cancer. *Cancer* **71**: 147–153

Cox DR (1972) Regression models and life-tables *JR Stat Soc* **84**: 1035–1044

Elston CW (1984) The assessment of histological differentiation in breast cancer. *Aust NZ J Surg* **54**: 11–15

Fukutomi T, Kobayashi Y, Nansawa T, Yamamoto H and Tsuda H (1993) A clinicopathological analysis of breast cancer patients with a family history. *Jpn J Surg* **23**: 849–854

Greenman J, Mohammed S, Ellis D, Watts S, Scott G, Izatt L, Barnes D, Solomon E, Hodgson S and Mathew C (1998) Identification of missense and truncating mutations in the *BRCA1* gene in sporadic and familial breast and ovarian cancer. *Genes Chromosomes Cancer* **21**: 244–249

Jacquemier J, Eisinger F, Birnbaum D and Sobul H (1995) Histoprognostic grade in BRCA1-associated breast cancer. *Lancet* **345**: 1503

Kaplan EL and Meier P (1985) Nonparametric estimation from incomplete observations. *Am Stat Assoc J* **53**: 457–481

Kelsell DP, Spurr NK, Barnes DM, Gusterson B and Bishop DT (1996) Combined loss of *BRCA1/2* in grade 3 breast carcinomas. *Lancet* **347**: 1554–1555

Letters to the Editor (1997) Cannistra SA, Modan B, Brunet J-S, Narod SA, Tonin PA, Foulkes WD and Rubin SC. *BRCA1* mutations and survival in women with ovarian cancer (letter). *N Engl J Med* **336**: 1254–1257

Lynch HT, Fain PR, Goldgar D, Albano WA, Mailliard JA and McKenna P (1981) Familial breast cancer and its recognition in the oncology clinic. *Cancer* **47**: 2730–2739

Malone KE, Daling JR, Weiss NS, McKnight B, White E and Voigt LF (1996) Family history and survival of young women with invasive breast carcinoma. *Cancer* **78**: 1417–1425

© Cancer Research Campaign 1998

Marcus JN, Watson P, Page DL, Narod S, Lenoir GM, Tonin P, Linder-Stephenson L, Salerno G, Conway TA and Lynch HT (1996) Hereditary breast cancer, pathobiology, prognosis and *BRCA1* and *BRCA2* gene linkage. *Cancer* **77**: 697–709

Peto R, Pike MC, Armitage P, Breslow NE, Cox DR, Howard SV, Mantel N, McPherson K, Peto J and Smith PG (1977) Design and analysis of randomized clinical trials requiring prolonged observation of each patient. II. Analysis and examples. *Br J Cancer* **35**: 1–39

Porter DE, Cohen BB, Wallace MR, Smyth E, Chetty U, Dixon JM, Steel CM and Carter DC (1994) Breast cancer incidence, penetrance and survival in probable carriers of *BRCA1* gene mutation in families linked to *BRCA1* on chromosome 17q21. *Br J Surg* **81**: 1512–1515

Rosen PP, Lesser ML, Senie RT and Kinne DW (1982) Epidemiology of breast carcinoma. III. Relationship of family history to tumour type. *Cancer* **50**: 171–179

Rubin SC, Benjamin I, Behbakht K, Takahashi H, Morgan MA, LiVolsi VA, Berchuck A, Moto MG, Garber JE, Weber B, Lynch HT and Boyd J (1996) Clinical and pathological features of ovarian cancer in women with germ-line mutations of *BRCA1*. *N Engl J Med* **335**: 1413–1416

*© Cancer Research Campaign 1998*